# Optimising adolescent health: a comparative study of high-intensity interval training and moderate-intensity continuous training on body composition and cardiovascular fitness in sedentary male youth

**DOI:** 10.3389/fspor.2025.1655906

**Published:** 2025-10-24

**Authors:** Huseyin Yahat

**Affiliations:** Faculty of Sport Sciences, Near East University, Nicosia, Cyprus

**Keywords:** adolescents, cardiorespiratory fitness, high-intensity interval training, moderate-intensity continuous training, sedentary lifestyle, weight management

## Abstract

**Background:**

Excess body fat and weight are key risk factors for morbidity and mortality, particularly during adolescence. High-Intensity Interval Training (HIIT) and Moderate-Intensity Continuous Training (MICT) are both widely used strategies to improve body composition, yet limited evidence exists comparing their effects among sedentary, normal-weight adolescent males.

**Methods:**

This randomized controlled study aimed to compare the effects of HIIT and MICT on body composition and cardiovascular fitness in sedentary male adolescents. Sixty normal-weight males aged 16–17 years were randomly assigned to one of three groups: HIIT (*n* = 20), MICT (*n* = 20), or control (CG; *n* = 20). The HIIT protocol comprised six 30-second high-intensity running intervals (80%–90% HRmax) interspersed with 90 s of low-intensity walking (50% HRmax), totalling 20 min per session. The MICT protocol involved continuous running at 60%–70% HRmax for 30 min, inclusive of warm-up and cool-down. Both intervention groups trained four times weekly over 8 weeks, while the control group received no intervention. Pre- and post-intervention measurements included body fat percentage, body weight, skinfold thickness, and resting heart rate, analysed using one-way ANOVA with Bonferroni *post hoc* comparisons. Given its shorter duration and comparable outcomes, HIIT appears time-efficient for school-based delivery in normal-weight adolescent males, addressing a population and setting under-represented in prior trials.

**Results:**

Significant reductions in body fat were observed in both the HIIT (−6.0%, *p* < 0.001, ES = 0.97) and MICT (−5.7%, *p* < 0.001, ES = 0.76) groups, with no meaningful change in the CG (−1.0%, *p* > 0.05). Both HIIT and MICT groups also demonstrated significant weight loss (−7.45%, *p* < 0.001), compared to a negligible change in CG (−0.89%, *p* > 0.05). Skinfold thickness significantly decreased in HIIT (−24.70%, *p* < 0.001) and MICT (−23.66%, *p* < 0.001), with minor change in CG (−4.12%, *p* > 0.05). Resting heart rate improved in HIIT (−9.14%, *p* < 0.001) and MICT (−7.12%, *p* < 0.001), whereas the CG experienced a slight increase (+0.026%, *p* > 0.05).

**Conclusions:**

Both HIIT and MICT are effective for improving body composition and cardiorespiratory fitness in sedentary male adolescents. Given its shorter duration and comparable outcomes, HIIT may be a time-efficient option for integration into school-based physical education

## 1 Introduction

Adolescent obesity is a pressing public health challenge. Affected youths face higher risks of morbidity and premature mortality than their normal-weight peers and are more likely to remain obese into adulthood (1, 2). Excess adiposity in childhood and adolescence is strongly linked to the early emergence of cardiovascular disease (3) and elevates the likelihood of type 2 diabetes (4, 5), stroke (6), and arterial stiffness (7) later in life. Because adolescence is a critical window for establishing lifelong health behaviours, regular physical activity is pivotal for limiting fat gain and supporting cardiovascular, metabolic, and mental health (8, 9).

Identifying the most effective exercise modalities is therefore essential. High-Intensity Interval Training (HIIT) has been reported to elicit greater excess post-exercise oxygen consumption (EPOC) and higher adherence than alternative protocols (10). The associated post-exercise metabolic elevation can augment fat oxidation and resting energy expenditure. HIIT is also time-efficient (11–13), making it well suited to school environments where curricular time is constrained. In this context, physical education (PE) classes offer a practical platform for integrating time-effective, physiologically potent interventions such as HIIT.

While PE in North Cyprus is traditionally delivered outdoors in school grounds, the present intervention was implemented in the National Sports Hall in Nicosia to standardise environmental conditions and ensure facility access. Delivery was supervised jointly by a lead researcher and participants' PE teachers, providing scientific oversight and pedagogical support. Framed as an extracurricular programme, the intervention intentionally shifted learning beyond conventional PE to promote sustainable, transformative experiences grounded in “learning by doing,” with an emphasis on experiential engagement and skill acquisition. The educational objective was to build students' understanding of how structured activity contributes to reductions in body fat and body weight, encouraging durable, health-promoting habits (14).

Despite growing interest, evidence directly comparing HIIT and Moderate-Intensity Continuous Training (MICT) for adolescent body-fat reduction remains limited and mixed (15–17). Prior studies often pooled heterogeneous age ranges (8–65 years), obscuring developmental differences (18–21). Physiological adaptations and substrate metabolism differ between children and adolescents (22), and sex-specific responses to HIIT are frequently under-reported. To enhance interpretability, the current study focused on male adolescents (23).

Notably, real-world applications remain underexplored, particularly in youth and young adults (11–13). An outdoor, 12-week programme in college students reported superior improvements with HIIT vs. MICT in cardiorespiratory fitness and body composition (24), and emerging evidence indicates that HIIT can be more time- and volume-efficient for cardiometabolic health in field conditions (25). These attributes reinforce HIIT's suitability for time-limited settings such as schools and community programmes (26).

A recent youth-focused meta-analysis restricted to overweight and obese samples between 9 and 19 years of reports that, vs. non-exercise controls, HIIT meaningfully reduces fat mass, waistline, body weight and diastolic blood pressure while markedly improving VO₂ max; in direct comparisons, HIIT exceeds MICT for VO₂ max and systolic blood pressure, with stronger effects in obese male adolescents and when frequency exceeds three sessions per week (27). These findings highlight efficacy but also the population boundary conditions (i.e., heavier youth) that limit generalization to normal-weight adolescents (28).

Complementing that synthesis, an RCT in obese adolescents contrasted aquatic vs. land-based HIIT (4 weeks, 3×/week). Both modes improved anthropometry, body fat percentage, blood pressure, and lipid markers, with aquatic HIIT additionally lowering resting heart rate and achieving greater gains in lean mass and ventilatory capacity (29). Although the study demonstrates feasibility and short-term responsiveness, its modest sample size, brief duration, and lack of an MICT or non-exercise comparator constrain inference about modality-specific advantages and longer-term cardiometabolic change (30).

Adult evidence provides additional context: a broad meta-analysis shows short- and long-term HIIT consistently increases VO₂ max, with more mixed or null effects on several traditional risk factors, particularly among normal-weight adults where VO₂ max improves but other outcomes often do not (11). This pattern cautions against assuming uniform transfer of body-composition or vascular benefits to normal-weight adolescents and supports prioritizing aerobic-capacity and adiposity endpoints while acknowledging heterogeneity across markers (31).

Adolescence is crucial for establishing lifelong health behaviours, yet evidence directly comparing HIIT and MICT in normal-weight adolescent males in school contexts remains sparse. Prior syntheses show HIIT and MICT can both improve adiposity and cardiometabolic markers, with mixed conclusions about superiority, and few studies isolate sex-specific responses in youth or consider time-efficiency relevant to PE timetables (32). This study addresses that gap by randomising sedentary, normal-weight male adolescents to HIIT, MICT, or control, implementing protocols compatible with school delivery, and benchmarking outcomes on body fat, weight, skinfolds, and resting heart rate with validated instruments.

Recent meta-analyses conclude that both interval training and moderate-intensity continuous training reduce adiposity, with interval formats yielding moderately greater absolute fat-mass losses in mixed samples of youth and adults (often overweight/obese) and across heterogeneous settings. In paediatric populations specifically, trials aggregated by García-Hermoso et al. (33) show that high-intensity interval protocols improve aerobic capacity and blood pressure more than comparison exercise in youths with overweight/obesity, again primarily outside tightly embedded school timetables. Collectively, these syntheses establish efficacy but leave two gaps: (i) limited randomized evidence in normal-weight adolescent males, among whom adiposity and cardiovascular fitness may still be suboptimal despite normative BMI; and (ii) a shortage of school-embedded, teacher-deliverable protocols that test time-efficiency under real scheduling constraints. The present trial was designed to address both gaps by randomising sedentary, normal-weight male adolescents within a secondary-school setting in the Eastern Mediterranean and by contrasting a 20-minute HIIT session with a 30-minute MICT session while standardising supervision, dosing, and measurement.

The aim of this study is to determine, in a randomised controlled, 8-week, school-based trial of sedentary, normal-weight male adolescents, whether high-intensity interval training (HIIT) produces greater reductions in whole-body adiposity than moderate-intensity continuous training (MICT) and a no-exercise control. The primary objective is change in body-fat percentage; secondary endpoints are changes in body weight, the sum of skinfolds, and resting heart rate. Intensity is prescribed by heart-rate reserve; interventions are delivered four times per week; outcomes are assessed pre/post with validated instruments and analysed with parametric tests. Therefore, the objectives are threefold.

• To compare pre-to-post change in body-fat percentage across HIIT, MICT and control.
• To compare pre-to-post changes in body weight, sum of skinfolds and resting heart rate across groups.
• To evaluate the time-efficiency and practical applicability of HIIT for integration into secondary-school physical-education delivery.

This study contributes four elements of originality: (i) a school-based, three-arm randomized comparison of HIIT, MICT, and control delivered four times weekly over eight weeks; (ii) a normal-weight male adolescent cohort, reducing confounding by excess adiposity prevalent in prior syntheses; (iii) a direct test of time-efficiency relevant to curriculum design; and (iv) standardized, blinded assessment of body-composition outcomes alongside resting heart rate as an index of cardiovascular fitness. These design choices target the practical translation of interval protocols into secondary-school physical education while complementing meta-analytic evidence derived largely from overweight/obese youths or non-school settings.

This randomised, 8-week, school-based trial highlights that high-intensity interval training (HIIT) produces a greater reduction in whole-body adiposity than moderate-intensity continuous training (MICT), with both exercise arms outperforming the no-exercise control. Beyond the primary endpoint (change in body-fat percentage), larger improvements are expected in secondary outcomes body mass, the sum of skinfolds, and resting heart rate in HIIT relative to MICT and control. Given shorter session duration at higher relative intensity, HIIT is anticipated to deliver comparable physiological benefits with less time, indicating time-efficiency for school implementation (34).

In this study, sedentary adolescents classified as normal-weight by BMI-for-age, HIIT will reduce whole-body adiposity (% body fat) more than MICT and control over 8 weeks. Because adolescents with normal-weight obesity can present with excess % body fat despite normal BMI, analyses were specified to target adiposity reduction, not ‘weight loss in overweight/obese youth (35).

The primary question asks whether, in sedentary, normal-weight male adolescents aged 16–17 years, an 8-week HIIT program reduces body-fat percentage more than MICT and more than a no-exercise control when intensity is prescribed using heart-rate reserve and training is delivered four times per week. Secondary questions examine between-group differences in pre-to-post changes in body mass, the sum of skinfolds, and resting heart rate, all assessed with validated instruments under a common measurement schedule. A translational question evaluates whether, considering session duration, HIIT constitutes a more time-efficient and school-feasible option than MICT for improving body composition and cardiorespiratory fitness in this population. In this study, the term cardiovascular fitness is used throughout to denote exercise-related functional capacity as reflected by resting heart rate in this study. No direct clinical markers of cardiovascular health (e.g., blood pressure, lipid profile, vascular function) were assessed; therefore, inferences are restricted to fitness adaptations.

### 1.1 Novelty of the study

Most adolescent HIIT evidence clusters around youths with overweight/obesity, feasibility narratives that are not tested inside real school schedules, or single-arm interventions without a direct comparator. Adult meta-analyses generalize robust VO₂ max benefits but do not speak to school delivery or to normal-weight teens, while recent youth syntheses prioritize moderator patterns in heavier cohorts. Against that backdrop, evidence isolating training-intensity effects in normal-weight male adolescents under conditions that mirror how schools could run sessions has remained unknown.

This study addresses the knowledge gap with a school-based, three-arm randomized design directly comparing HIIT, MICT, and a true control in sedentary, normal-weight male adolescents across 8 weeks at four sessions per week. The study identifies body-fat percentage as the primary endpoint alongside the sum of skinfolds, body mass, and resting heart rate assessed with validated tools under standardized, blinded procedures. By holding setting, supervision, and measurement constant while varying intensity prescription (HRR-based HIIT vs. MICT), the design delivers a clean head-to-head test that has been largely missing from the youth literature.

A second innovation is the explicit test of time-efficiency central to PE adoption. HIIT is implemented in 20-minute sessions and contrasted with 30-minute MICT, allowing the manuscript to speak to benefit per unit time a decision variable for schools balancing curricular time, staffing, and facility constraints. The intervention is timetabled in PE-compatible blocks (after-school/one weekend slot, more than 24-hour recovery), includes operational detail, and is accompanied by intensity-monitoring criteria. This moves beyond feasibility rhetoric to tested practice. It must be noted that by focusing on a male, normal-weight adolescent cohort, the study contributes sex- and weight-status–specific evidence that complements prior findings in heavier youth. This helps disentangle training responses from excess-adiposity confounding and speaks to the under-recognized “normal-weight, high-fat” risk profile seen in sedentary teens.

## 2 Methodology

### 2.1 Research design

This study used a three-arm randomized controlled design over 8 weeks. Participants were 60 sedentary, normal-weight male adolescents (16–17 years) allocated to HIIT (*n* = 20), MICT (*n* = 20), or control (*n* = 20). The HIIT protocol comprised six 30-s running bouts at 80%–90% HRmax interspersed with 90-s walking (50% HRmax), delivered four times per week and lasting 20 min including warm-up and cool-down. The MICT protocol involved continuous running at 60%–70% HRmax, also four times per week, lasting 30 min including warm-up and cool-down. The control group undertook no structured exercise. Exercise intensity was prescribed using the Karvonen heart-rate–reserve method. It must be noted that supervising staff observed participants during all sessions and instructed them to report any injury or illness; any event prompting session cessation, first-aid provision, or medical referral was predefined as an adverse event (36).

The primary outcome was body-fat percentage; secondary outcomes were body mass, skinfold thickness, and resting heart rate. Measurements were obtained with validated instruments (InBody 770 for %fat; Holtain calipers under ISAK procedures for skinfolds; Polar V800 for heart rate) between 09:00 and 11:00 under standardized conditions by assessors blinded to group. The trial did not impose caloric restriction. A dietitian provided a standardized meal pattern (food lists/portion guidance; 2,500–3,000 kcal·day^−1^ appropriate for adolescent males). Participants were instructed to keep their usual pattern stable and to avoid new dietary practices. Adherence was monitored weekly via a Diet & Lifestyle Checklist (see Appendix A). Because intake was not quantified, outcomes were not adjusted for energy intake; this is acknowledged as a limitation. Diet was standardized via a prescribed meal pattern and adherence checklist; however, energy intake was not quantified, leaving the possibility of residual confounding (37). In-session heart-rate data were summarized at the group level, and incomplete coverage in the MICT arm. Overall, the findings reflect an extracurricular program that supplements rather than replaces physical education; the impact of long-term curricular integration therefore warrants separate evaluation.

The control group received no structured exercise and was instructed to maintain usual routines. Dietary guidance (standardised meal pattern and “keep usual pattern stable”) and a weekly Diet & Lifestyle Checklist were provided across groups to minimise differential co-interventions; however, energy intake was not quantified. Free-living physical activity in the control arm was not objectively monitored (e.g., accelerometry), and session-level adherence/fidelity metrics were collected only for the exercise arms and were incomplete in parts of MICT. These features are acknowledged as potential sources of residual confounding.

It must be noted that height and body mass were measured using standardized procedures and BMI was calculated as mass (kg)/height (m^2^). BMI-for-age z-scores and percentiles were derived using the both WHO 2007 and CDC 2000 benchmarking criteria reference with age in months and sex-specific LMS parameters; weight status was classified as <5th (underweight), 5th–<85th (normal-weight), 85th–<95th (overweight), and ≥95th percentile (obese).

### 2.2 Study participants

Sixty sedentary, normal-weight male adolescents aged 16–17 years participated in this study.

Sedentary status was defined *a priori* as International Physical Activity Questionnaire–Short Form (IPAQ-SF) < 600 MET-min·week^−1^ (low activity). All enrolled participants met this criterion at screening. From an initial recruitment of 74 volunteers, 60 students who met the eligibility criteria were selected and randomly assigned to one of three groups: High-Intensity Interval Training (HIIT), Moderate-Intensity Continuous Training (MICT), or a non-intervention control group (CG), with 20 participants in each. The mean age across all groups was 16.10 ± 0.30 years. At baseline, the HIIT group had a mean body fat percentage of 26.00 ± 2.97%, body weight of 77.15 ± 4.39 kg, skinfold thickness of 24.90 ± 2.91%, and resting heart rate of 78.25 ± 3.30 bpm. The MICT group presented with a mean body fat percentage of 25.80 ± 3.03%, body weight of 77.20 ± 5.01 kg, skinfold thickness of 24.30 ± 2.79%, and resting heart rate of 80.05 ± 3.57 bpm. The control group recorded a mean body fat percentage of 25.20 ± 3.05%, body weight of 79.10 ± 4.11 kg, skinfold thickness of 23.05 ± 3.08%, and resting heart rate of 78.45 ± 1.43 bpm. Baseline measurements confirmed homogeneity across the three groups, ensuring comparability prior to the intervention (38).

**Figure 1:**
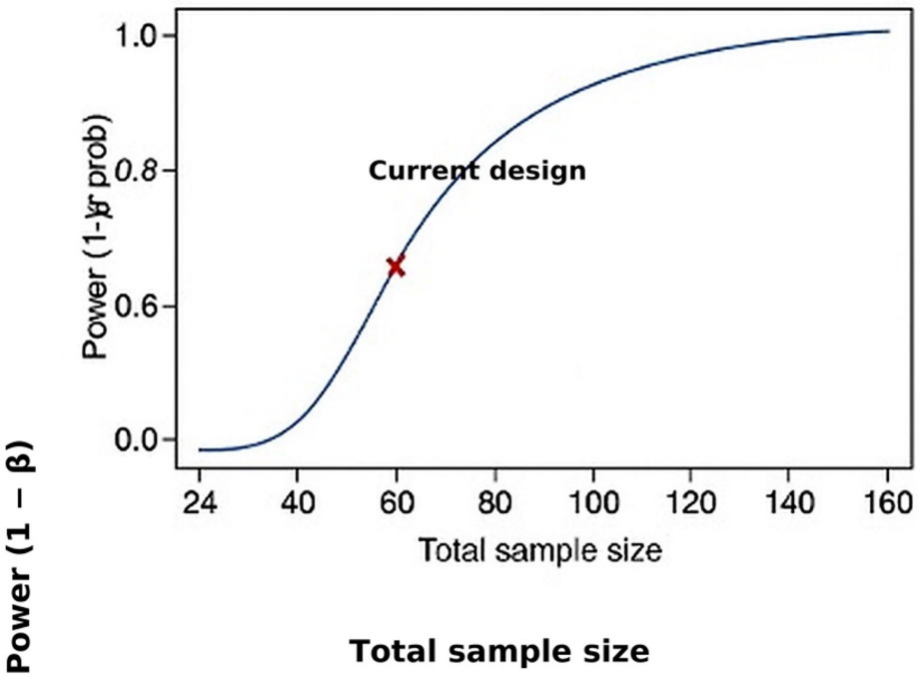
*a priori* power analysis using G*Power* 3.1.9.7 indicating the minimum required sample size (*n* = 24) to detect a medium effect size (*f* = 0.25) with 80% power at *α* = 0.05. Each study group (HIIT, MICT, and Control) included 20 participants, exceeding the minimum requirement of 8 participants per group.

As shown in Figure 1, *a priori* sample-size estimation with G*Power indicated a minimum of 24 participants (effect size f = 0.25, *α* = 0.05, power 1–*β* = 0.80); the final sample comprised 60 (20 per group). Statistical analyses included assumption checks (Shapiro–Wilk for normality, Levene's test for homogeneity), followed by one-way ANOVA with Bonferroni-adjusted *post hoc* comparisons (*α* = 0.05). Effect sizes were expressed as partial *η*^2^, with thresholds of 0.01 (small), 0.059 (moderate), and 0.138 (large).

### 2.3 Intervention

**Figure 2:**
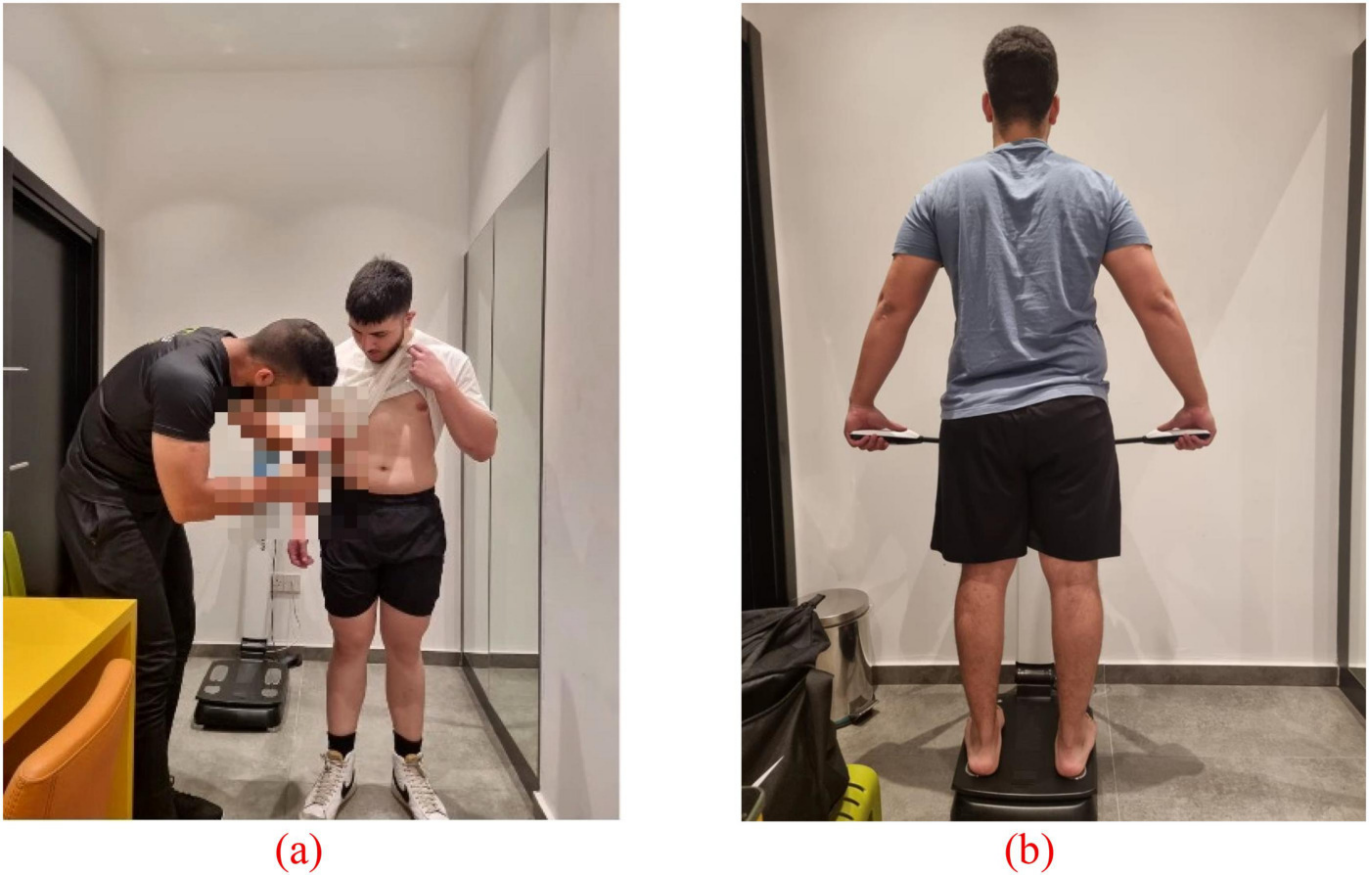
**(a)** estimating body fat using skinfold thickness calipers; **(b)** body fat percentage assessment via bioelectrical impedance analysis.

Training was delivered as an extracurricular program in the National Sports Hall under joint supervision of the first researcher and school PE staff. Each intervention arm completed four sessions per week with a minimum of 24 h between sessions; no back-to-back sessions were scheduled on school days. A typical weekly roster was Monday-Wednesday-Friday during after school hours and Sunday morning, with HIIT and MICT run in staggered 30–35-min blocks (separate start times) to avoid crowding and to ensure equivalent facility access. Exercise intensity was prescribed using heart-rate reserve (HRR; Karvonen) individualized to each participant (39). Heart rate was recorded continuously at 5-s epochs (Polar devices) during all sessions. Compliance was defined *a priori* as more than 80% of work-interval time within the target zone (HIIT: 80%–90% HRmax during 30-s bouts; MICT: continuous 60%–70% HRmax), as shown in Figures 2a,b.

The program was approved by the school administration and implemented outside regular PE lessons; PE curricula were unaffected. All participants provided written parental/guardian consent and student assent (40). The intervention is presented as a supplement to PE, not a replacement. The study seeks to operationalise sedentary status: eligibility required the IPAQ-SF low-activity classification (more than 600 MET-min·week^−1^) following PAR-Q screening, and all randomized participants met this threshold. However, no descriptive IPAQ-SF screening outputs are presented—such as total MET-min·week^−1^ by group.

Body composition was assessed using body mass and summed skinfolds because these indices are widely used, low-burden, and responsive over 6–8 weeks in adolescent interventions; measurements were standardised (timing, devices, assessor blinding) to enhance reliability. Cardiovascular fitness was indexed by resting heart rate as a pragmatic surrogate of autonomic and training status suitable for repeated assessment within PE-compatible schedules. These endpoints balanced methodological rigour with operational constraints (session length, staffing, facility access), enabling high-frequency data collection without disrupting the school timetable.

It has to be noted that both the HIIT and MICT groups served as the intervention groups and participated in structured exercise sessions four times per week on non-consecutive days for a duration of eight weeks. An 8-week intervention period is consistent with previous HIIT studies and has been demonstrated to yield measurable physiological adaptations (3, 19). Training intensity for both groups was individually prescribed using the Karvonen formula, a widely recognized method for determining target heart rate zones in exercise programming (41). The HIIT group performed a protocol consisting of six cycles of 30 s of high-intensity running at 80%–90% of maximum heart rate (HRmax), followed by 90 s of low-intensity walking until heart rate decreased to approximately 50% of HRmax. Each session lasted 20 min in total, including warm-up and cool-down periods.

**Figure 3:**
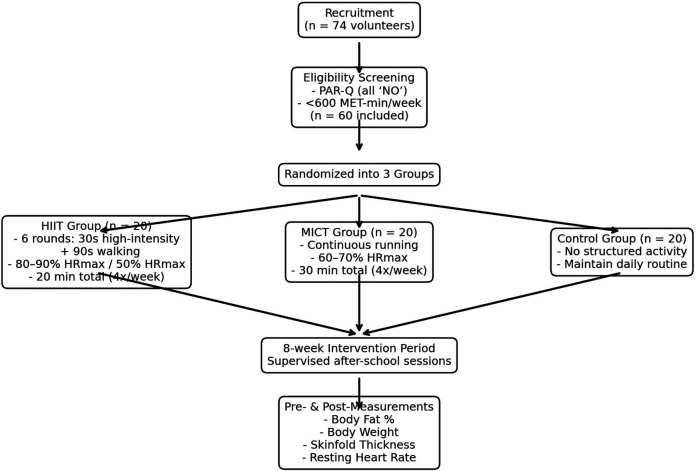
Step-by-step development of the methodological framework.

Figure 3 demonstrates the participant recruitment, group allocation, and intervention protocol. A total of 74 volunteers were screened, and 60 eligible sedentary, normal-weight male adolescents (based on PAR-Q and <600 MET-min/week) were randomly assigned to three groups: HIIT (*n* = 20), MICT (*n* = 20), and Control (*n* = 20). Intervention groups completed structured exercise sessions four times per week for 8 weeks, while the control group maintained usual routines. Pre- and post-intervention assessments included body fat percentage, body weight, skinfold thickness, and resting heart rate. Table 1 delineates the experiment parameters for the study.

**Table 1:** Summary of intervention protocols and outcome measures for the HIIT, MICT, and control groups, including exercise structure, duration, frequency, and assessed physiological parameters.

Group	Sample size (n)	Exercise description	Session duration	Frequency	Warm-up & cool-down	Measurement parameters
HIIT	20	6 rounds: 30s high-intensity running + 90s walking (80%–90% HRmax/50% HRmax)	20 min	4 sessions/week	Included (5–6 min)	Body fat %, body weight, skinfold thickness, resting heart rate
MICT	20	Continuous running at 60%–70% HRmax	30 min	4 sessions/week	Included (5–6 min)	Body fat %, body weight, skinfold thickness, resting heart rate
Control	20	No structured activity; usual routine	—	—	—	Body fat %, body weight, skinfold thickness, resting heart rate

The MICT group engaged in continuous moderate-intensity running, maintaining 60%–70% of HRmax for a total of 30 min per session, also inclusive of warm-up and cool-down phases. Warm-up exercises (5–6 min) included light jogging and dynamic stretching movements such as leg swings and high knees. Cool-down exercises (5–6 min) consisted of static stretching routines to facilitate recovery and flexibility. The Control Group (CG) did not participate in any structured physical activity during the intervention period. Participants in this group were instructed to maintain their normal daily routines and refrain from engaging in any additional exercise beyond their usual behaviour.

### 2.4 Data collection

Data were collected at baseline and following the 8-week intervention period to enable pre- and post-intervention comparisons. Total body fat percentage and body weight were assessed using the InBody 770*,* a high-end bioelectrical impedance analysis (BIA) device recognized for its precision in evaluating body composition (42). Skinfold thickness was measured using the Holtain Skinfold Caliper*,* a research-grade instrument widely acknowledged for its high accuracy and reliability in anthropometric assessments. Resting heart rate was recorded using the *Polar V800 heart rate monitor* (Polar Electro Oy Inc., Kempele, Finland), a validated device commonly used in sports and exercise science research (43). While the primary outcome variable was the change in total body fat percentage, secondary outcomes included changes in body weight, skinfold thickness, and resting heart rate, all of which are physiologically relevant markers associated with fat reduction and cardiometabolic health (44).

**Figure 4:**
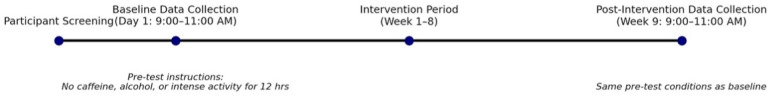
Data collection timeline for the study.

As shown in Figure 4, to ensure the reliability of measurements, all assessments were conducted between 9:00 AM and 11:00 AM under standardized conditions. Participants were instructed to abstain from caffeine, alcohol, and strenuous physical activity for at least 12 h prior to testing (45). The third and fourth researchers were responsible for ensuring the regular calibration of all devices, following manufacturer guidelines to maintain measurement accuracy and internal validity. To minimize measurement bias, the researchers responsible for data collection were blinded to group allocation. Participants were assigned neutral identifiers (Group A, B, or C), and the data collectors was not involved in administering the intervention. To assess the success of the blinding procedure, a post-assessment survey was conducted in which the researchers were asked to guess each participant's group. The correct identification rate was 55%, indicating an adequate level of blinding.

Adherence and fidelity were prespecified outcomes to contextualise training responsiveness. Session attendance (proportion of the 32 scheduled sessions completed) and session-level heart-rate capture for time-in-zone (%HRR within target ranges) were the planned indices. Participant flow (allocation, retention, analysis set) and dropout numbers with reasons were recorded following CONSORT principles. Where heart rate or attendance logs were incomplete, adherence was not estimated for those cases.

Skinfold measurements were conducted following the standards of the International Society for the Advancement of Kinanthropometry (ISAK). Measurements were taken at four anatomical sites commonly used for male participants: the biceps, triceps, subscapular, and iliac crest (46). All assessments were conducted on the right side of the body to ensure consistency. Two trained researchers performed multiple measurements on each participant under the same conditions, and the median value was used to improve precision and reduce random error. To minimize inter-tester variability, the same skinfold calliper was used throughout the study, and all measurements were conducted with careful adherence to ISAK protocols, as shown in Table 2.

**Table 2:** Reliability assessment of skinfold thickness measurements showing high intra- and inter-rater agreement, confirming measurement consistency and methodological rigor across both raters.

Assessment type	Rater (s)	Correlation coefficient (r)
Intra-rater Reliability	Rater 1	0.971
Intra-rater Reliability	Rater 2	0.967
Inter-rater Reliability	Rater 1 vs Rater 2 (mean values)	0.988

To ensure the reliability of skinfold thickness measurements, both intra- and inter-rater agreement analyses were conducted. Intra-rater reliability was assessed by comparing repeated measurements taken by the same rater under identical conditions (47). The intra-rater reliability coefficients were *r* = 0.971 for Rater 1 and *r* = 0.967 for Rater 2, indicating excellent internal consistency. Inter-rater reliability was evaluated by correlating the average skinfold values obtained independently by both raters. The inter-rater correlation coefficient was *r* = 0.988, reflecting very high agreement between raters. These results support the precision and consistency of the anthropometric measurements used in this study.

### 2.5 Statistical analysis

**Figure 5:**
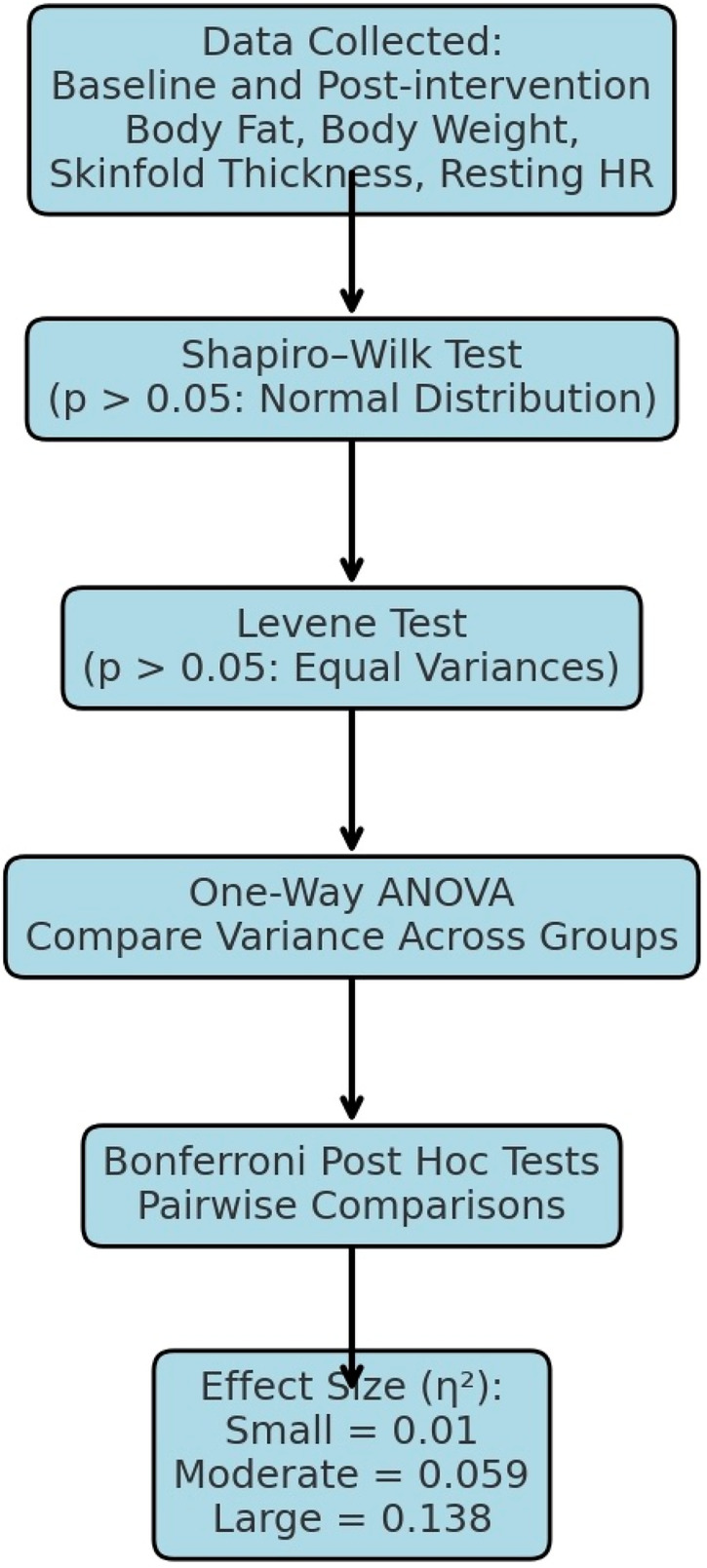
Statistical method developed for the study.

Statistical analyses were conducted on the baseline and post-intervention values for body fat percentage, body weight, skinfold thickness, and resting heart rate (48). Normality of data distribution was assessed using the Shapiro–Wilk test, which is considered one of the most robust tests for detecting deviations from normality (49). Homogeneity of variances across groups was evaluated using the Levene test. The results confirmed that all data met the assumptions of normality and homogeneity, with *p* > 0.05 for each variable and group, as shown in Figure 5.

Following confirmation of parametric assumptions, a one-way Analysis of Variance (ANOVA) was employed to identify statistically significant differences among the three groups (HIIT, MICT, and control) for each outcome variable (42). Where significant group effects were detected, Bonferroni *post hoc* tests were applied to determine pairwise differences while controlling for Type I error. The threshold for statistical significance was set at *p* < 0.05. For each statistically significant finding, effect sizes (ES)were calculated using partial eta-squared (*η*^2^) to interpret the magnitude of the effects. The following classification criteria were applied: small (*η*^2^ = 0.01), moderate (*η*^2^ = 0.059), and large (*η*^2^ = 0.138).

Effect magnitudes are reported quantitatively rather than by qualitative labels. For each omnibus ANOVA, partial *η*p^2^ with 95% confidence intervals (CIs) was derived from the non-central F distribution. For pairwise contrasts and change-score comparisons, Hedges g (or mean differences) with 95% CIs and exact *p*-values are presented. For all primary and secondary outcomes (body-fat percentage, body mass, summed skinfolds, resting heart rate), tables provide pre, post, and Δ [mean (95% CI)] by group, plus between-group *Δ* differences with 95% CIs. Where model assumptions were not met, the appropriate alternative model and its effect-size analogue are specified. Verbal descriptors such as “small/moderate/large” are avoided unless accompanied by the corresponding statistic and CI.

## 3 Analysis and results

### 3.1 Baseline

Participants were randomly assigned (1:1:1) to HIIT, MICT, or control using a computer-generated sequence. Allocation was concealed until assignment with sequentially numbered, opaque, sealed envelopes prepared by an administrator independent of enrolment and outcome assessment. Group balance at baseline is shown in Tables 3, 4. Participant retention was complete across arms over 8 weeks (HIIT *n* = 20, MICT *n* = 20, Control *n* = 20; 0% attrition; no withdrawals or losses to follow-up). Session-level heart-rate capture was incomplete in parts of the MICT arm, and comprehensive attendance proportions were not available for all participants; consequently, adherence rates (% sessions completed) are not reported. Any efficiency comparisons are therefore presented without compliance adjustment.

**Table 3:** Baseline characteristics of variables for the statistical analysis.

Group	Body weight (kg)	Body fat (%)	Skinfold (mm)	Resting HR (bpm)
HIIT	77.15 ± 4.39	26.00 ± 2.97	24.90 ± 2.91	78.25 ± 3.30
MICT	77.20 ± 5.01	25.80 ± 3.03	24.30 ± 2.79	80.05 ± 3.57
Control	79.10 ± 4.11	25.20 ± 3.05	23.05 ± 3.08	78.45 ± 1.43

**Table 4:** Baseline anthropometrics of total sample size for the study.

Variables	Values
Height (cm), mean ± SD	176.05 ± 4.22
Height (cm), median [IQR]	176.0 [173.0–179.0]
Weight (kg), mean ± SD	77.82 ± 4.54
Weight (kg), median [IQR]	79.0 [75.0–81.2]
BMI (kg/m^2^), mean ± SD	25.15 ± 1.86
BMI (kg/m^2^), median [IQR]	25.22 [23.80–26.21]
BMI (kg/m^2^), min–max	20.45–29.76
QC: BMI bands (adult cutoffs)	18.5–24.9: 28; 25.0–29.9: 32; < 18.5: 0; ≥ 30: 0

**Table 5:** Between-group differences in change: body Fat (%) (r = 0.50).

Comparison	Mean *Δ*	95% CI low	95% CI high	Hedges g
HIIT – Control	−5.00%-points	−6.91	−3.09	−1.64
MICT – Control	−4.70%-points	−6.70	−2.70	−1.47
HIIT – MICT	−0.30%-points	−2.37	1.77	−0.09

The baseline study was homogeneous in stature and mass, with a mean height of 176.05 ± 4.22 cm and body mass of 77.82 ± 4.54 kg, yielding a mean BMI of 25.15 ± 1.86 kg m^−2^ (median [IQR] 25.22 [23.80–26.21]; range 20.45–29.76). A simple quality check against adult BMI bands indicated that 28 of 60 participants fell within 18.5–24.9 kg m^−2^ and 32 of 60 within 25.0–29.9 kg m^−2^, with none underweight or class I obese, as shown in Tables 3, 4.

Randomisation achieved close between-group comparability across key anthropometric and physiological variables. Baseline body mass was 77.15 ± 4.39 kg (HIIT), 77.20 ± 5.01 kg (MICT), and 79.10 ± 4.11 kg (Control). Whole-body adiposity was similar—26.00 ± 2.97%, 25.80 ± 3.03%, and 25.20 ± 3.05% for HIIT, MICT, and Control, respectively—as were skinfold sums (24.90 ± 2.91, 24.30 ± 2.79, 23.05 ± 3.08 mm) and resting heart rate (78.25 ± 3.30, 80.05 ± 3.57, 78.45 ± 1.43 bpm). Absolute differences were small and within approximately one SD, indicating no material baseline imbalance likely to confound treatment effects; this can be confirmed with one-way ANOVA (or Kruskal–Wallis if distributional assumptions are violated). Given the BMI distribution near the adult overweight threshold alongside mean baseline body-fat values of 25%–26%, the trial is appropriately positioned to target adiposity change rather than generic weight loss as shown in Table 5.

Tables between 6, 7 show the baseline parameters by using change-score re-analysis with SD of change computed as

**Table 6:** Between-group differences in change: body weight (kg) (r = 0.50).

Comparison	Mean *Δ*	95% CI low	95% CI high	Hedges g
HIIT – Control	−5.05 kg	−7.65	−2.45	−1.22
MICT – Control	−5.05 kg	−7.82	−2.28	−1.14
HIIT – MICT	0.00 kg	−2.91	2.91	0.00

**Table 7:** Estimated fat mass change (kg) from group means.

Group	Fat mass (kg) Pre	Fat mass (kg) Post	*Δ* Fat mass (kg)
HIIT	20.06	14.28	−5.78
MICT	19.92	14.36	−5.56
Control	19.93	18.97	−0.96

SD*_Δ_* = √ (SD_pre_^2^ + SD_post_^2^−2r SD_pre_ SD_post_) (primary assumption *r* = 0.50), HIIT and MICT produced substantially greater reductions in whole-body fat than control. The mean difference in change was −5.00%-points for HIIT vs. control (95% CI −6.91 to −3.09; Hedges g = −1.64) and −4.70%-points for MICT vs. control (95% CI −6.70 to −2.70; g = −1.47); HIIT and MICT did not differ (−0.30%-points; 95% CI −2.37 to 1.77; g = −0.09). For body mass, both training conditions showed −5.05 kg greater loss than control (HIIT vs. control 95% CI −7.65 to −2.45, g = −1.22; MICT vs. control 95% CI −7.82 to −2.28, g = −1.14), with no difference between HIIT and MICT (0.00 kg; 95% CI −2.91 to 2.91; g = 0.00).

A similar pattern emerged for body mass. Both HIIT and MICT achieved markedly greater weight loss than control (each −5.05 kg; HIIT 95% CI −7.65 to −2.45; MICT 95% CI −7.82 to −2.28), with no difference between the two active arms (0.00 kg; 95% CI −2.91 to 2.91). Translating the percentage changes to absolute fat mass for clinical interpretation, estimated fat mass decreased by 5.78 kg in HIIT and 5.56 kg in MICT, compared with 0.96 kg in the control group.

### 3.2 Intervention values

Prior to the intervention, baseline measurements were conducted to assess potential effect modifiers, including body fat percentage, body weight, skinfold thickness, resting heart rate, and chronological age. These variables were statistically compared across the three groups HIIT, MICT, and control (CG) to ensure initial group equivalence. No statistically significant differences were found in any of the pre-intervention variables (*p* > 0.05), confirming the homogeneity of the groups at baseline (50).

The mean body fat percentage was 26.00 ± 2.97% for the HIIT group, 25.80 ± 3.03% for the MICT group, and 25.20 ± 3.05% for the CG. The mean body weight was 77.15 ± 4.39 kg for HIIT, 77.20 ± 5.01 kg for MICT, and 79.10 ± 4.11 kg for CG. For skinfold thickness, the average values were 24.90 ± 2.91% in the HIIT group, 24.30 ± 2.79% in the MICT group, and 23.05 ± 3.08% in the CG. The resting heart rate averages were 78.25 ± 3.30 bpm for HIIT, 80.05 ± 3.57 bpm for MICT, and 78.45 ± 1.43 bpm for CG. Across all groups, the mean age was identical at 16.10 ± 0.30 years. These findings confirmed that the groups were statistically comparable prior to the intervention. A detailed summary of the pre- and post-intervention comparisons of body fat percentage, body weight, skinfold thickness, and resting heart rate across all groups is presented in Table 8.

**Table 8:** Comparison of pre-intervention and post-intervention values of all groups.

Variables		Pre-intervention		Post-intervention	
	Group	x¯	Ss	x¯	Ss
Body fat (%)	HIIT	26.00	2.97	20.00	3.21
	MICT	25.80	3.03	20.10	3.62
	CG	25.20	3.05	24.20	2.66
Body weight (kg)	HIIT	77.15	4.39	71.40	4.18
	MICT	77.20	5.01	71.45	4.53
	CG	79.10	4.11	78.40	3.45
Skinfold caliper (mm)	HIIT	24.90	2.91	18.75	3.12
	MICT	24.30	2.79	18.55	3.45
	CG	23.05	3.08	22.10	3.07
Resting heart rate (bpm)	HIIT	78.25	3.30	71.10	3.07
	MICT	80.05	3.57	74.35	3.57
	CG	78.45	1.43	78.65	1.98

Effect sizes classified as small (0.01), moderate (0.059) or large (0.138).

The results of the statistical analyses indicate significant within-group improvements in all physiological parameters for the intervention groups (HIIT and MICT), with negligible changes observed in the control group. With respect to body fat percentage, the HIIT group exhibited a reduction from 26.00 ± 2.97% to 20.00 ± 3.21%, while the MICT group showed a comparable decrease from 25.80 ± 3.03% to 20.10 ± 3.62%. In contrast, the control group demonstrated only a marginal reduction from 25.20 ± 3.05% to 24.20 ± 2.66%. These findings suggest that both exercise modalities significantly contributed to fat loss, with effect sizes falling within the “large” range as per partial eta-squared classification (51).

In terms of body weight, both the HIIT and MICT groups experienced substantial and nearly identical reductions. The HIIT group decreased from 77.15 ± 4.39 kg to 71.40 ± 4.18 kg, and the MICT group from 77.20 ± 5.01 kg to 71.45 ± 4.53 kg. The control group showed only a minor decrease from 79.10 ± 4.11 kg to 78.40 ± 3.45 kg. These outcomes reinforce the efficacy of both HIIT and MICT interventions in promoting weight reduction. Regarding skinfold thickness**,** the HIIT group reduced from 24.90 ± 2.91% to 18.75 ± 3.12%, and the MICT group from 24.30 ± 2.79% to 18.55 ± 3.45%. The control group's values shifted slightly from 23.05 ± 3.08% to 22.10 ± 3.07%. The notable declines in both intervention groups, compared with the minimal change in the control group, indicate meaningful improvements in subcutaneous fat levels attributable to structured exercise. For resting heart rate, the HIIT group demonstrated a decrease from 78.25 ± 3.30 bpm to 71.10 ± 3.07 bpm, whereas the MICT group reduced from 80.05 ± 3.57 bpm to 74.35 ± 3.57 bpm. Conversely, the control group showed a slight increase from 78.45 ± 1.43 bpm to 78.65 ± 1.98 bpm. These findings highlight significant cardiovascular benefits in both intervention groups, with the HIIT group exhibiting a marginally greater improvement (52).

In summary, the findings affirm the efficacy of both HIIT and MICT in improving body composition and cardiovascular fitness among sedentary, normal-weight male adolescents. The observed changes across all variables, thereby supporting the study's hypothesis and emphasizing the clinical relevance of these interventions in school-based physical activity programs.

### 3.3 Heart rate

In this study, participant retention was complete across arms (0% attrition/dropout) during the 8-week period. However, adherence indices session attendance proportions and heart-rate time-in-zone (%HRR) were not comprehensively quantified for all participants, and session-level heart-rate capture was incomplete in parts of the MICT arm. Consequently, efficiency comparisons are presented without compliance adjustment. Session prescriptions differed nominally in planned duration (HIIT = 20 min; MICT = 30 min, both inclusive of warm-up/cool-down). Training heart rate was analysed using within-subject intensity (65% vs. 85%) and between-group (HIIT vs. MICT) factors. Because the Control group lacked paired intensity readings in the provided dataset, the mixed model was restricted to groups with complete pairs. ΔHR (85–65 bpm) was computed per participant. A one-way ANCOVA on *Δ*HR compared groups (HIIT, MICT) while adjusting for Height and baseline resting HR (mean of three pre-trial readings). Within-group intensity effects were evaluated by paired *t*-tests (85 vs. 65). Descriptives are reported as means ± SD. Significance was set at *α* = 0.05.

**Figure 6:**
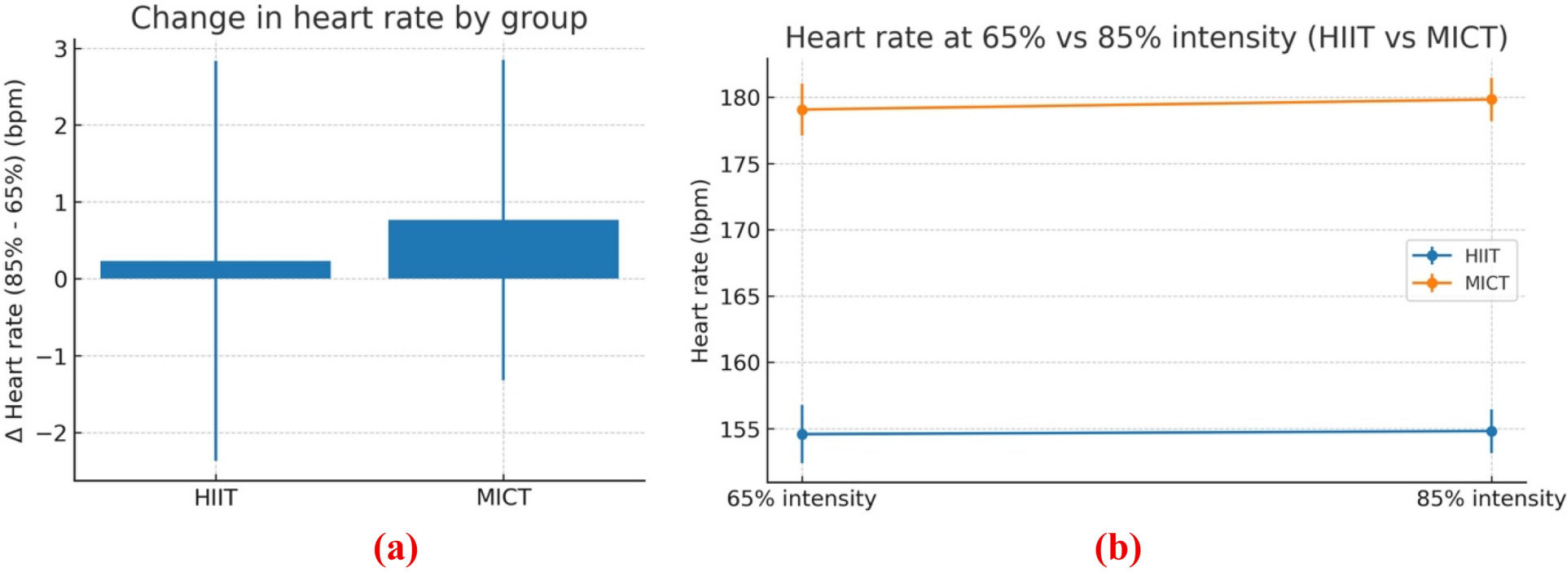
**(a)** change in training heart rate (85%–65%) by group (HIIT vs MICT); **(b)** heart rate at prescribed 65% and 85% intensities for HIIT and MICT (mean ± SD).

Figure 6a shows the within-subject change in training heart rate from 65% to 85% intensity (Δ = 85%–65%) for the two exercise groups. Mean deltas are trivial: + 0.2 bpm in HIIT and +0.8 bpm in MICT, with very wide variability. The substantial overlap and large dispersion indicate that neither group shows a reliable increase in heart rate when moving from the 65% to the 85% session. In practical terms, the two prescribed intensities were not physiologically separated during execution. This is consistent with non-significant paired tests (HIIT *p* = 0.69; MICT *p* = 0.28) and a non-significant between-group comparison of *Δ* after covariate adjustment in our earlier analyses. The pattern suggests either (i) participants often failed to reach/maintain the target 85% zone, (ii) the 65% sessions drifted higher than prescribed, and/or (iii) incomplete HR capture diluted estimates. For reporting, interpret the manipulation check as not met, and include session-level HR compliance (e.g., % time-in-zone) and procedures to improve fidelity (real-time HR feedback, staggered starts, coach prompts, repeat sessions when out of zone).

Figure 6b compares mean training heart rate at the prescribed 65% vs. 85% intensity for HIIT and MICT (error bars = SD). Values are almost flat within each group: HIIT 154.6 ± 2.2 vs. 154.8 ± 1.7 bpm, MICT 179.1 ± 2.0 vs. 179.8 ± 2.2 bpm. These trivial changes (+0.2–0.8 bpm) are tiny relative to the within-group variability, and paired tests are non-significant (HIIT *p* = 0.69; MICT *p* = 0.28). Thus, the intended intensity manipulation did not produce higher heart rates at 85% than at 65%, indicating poor separation of workloads during execution. The consistent between-group offset (MICT = 25 bpm higher than HIIT at both time points) reflects different absolute speeds/contexts rather than a true intensity contrast and should not be interpreted as superior effort. Practically, treat this as a failed manipulation check: conclusions about “85% vs. 65%” effects are unsupported. For fidelity, report time-in-zone (%HRR) per session, use real-time HR feedback and coach prompts, standardize warm-ups and recovery, and consider re-classifying sessions by actual %HRR achieved rather than prescription labels (53).

As shown in Table 9, an ANCOVA on ΔHR (85%–65%) with Height and baseline resting HR as covariates showed no between-group differences [F(2, ·) = 2.56, *p* = 0.097]; Height was a significant covariate [F(1, ·) = 5.50, *p* = 0.027], and baseline resting HR trended toward significance [F(1, ·) = 3.17, *p* = 0.087].

**Table 9:** ANCOVA analysis between HIIT and MICT.

Variables	sum_sq	df	F	PR (>F)
Group	27.24502	2	2.561481	0.096516482
Height	29.24493	1	5.49901	0.026936278
RestHR_pre_mean	16.85511	1	3.169315	0.086724639

As shown in Table 10, paired *t*-tests showed no significant within-group increase in training heart rate when moving from 65% to 85% intensity. In HIIT (*n* = 20), the mean change was +0.23 bpm, *t*(19) = 0.401, *p* = .693 (95% CI −0.99 to +1.45; trivial effect, *d* = 0.09). In MICT (*n* = 10), the mean change was +0.77 bpm, *t*(9) = 1.163, *p* = 0.275 (95% CI −0.72 to +2.26; small effect, *d* = 0.37). Confidence intervals include zero in both groups, confirming that the intended intensity separation was not physiologically achieved. Reductions in resting heart rate indicate improved cardiovascular fitness, with larger mean decreases in HIIT than in MICT and negligible change in controls. These results support treating the manipulation check as failed and, for fidelity, reporting time-in-zone (%HRR) and/or reclassifying sessions by actual %HRR achieved rather than prescribed labels.

**Table 10:** Paired *t*-test between HIIT and MICT.

Group	n	mean_65	mean_85	delta_mean	t	P
HIIT	20	154.5667	154.8	0.233333	0.400957	0.692925
MICT	10	179.0667	179.8333	0.766667	1.16283	0.274803

### 3.4 Body composition

**Figure 7:**
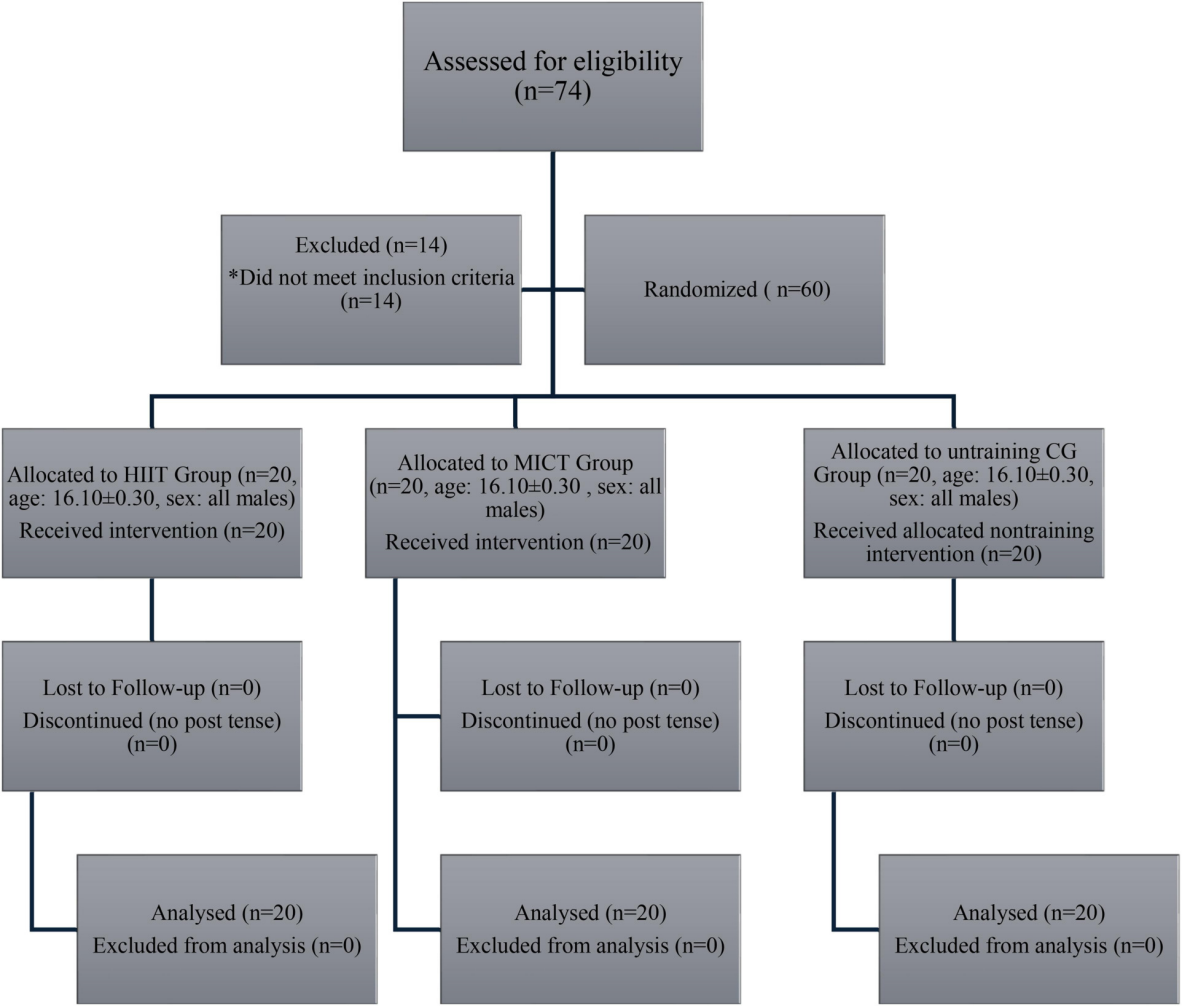
CONSORT flow diagram showing participant allocation, retention, and analysis across HIIT, MICT, and control groups.

Within-group analyses revealed statistically significant reductions in the HIIT group across all primary outcome measures. Specifically, body fat percentage decreased significantly (*p* < 0.001, ES = 0.97, 95% CI), as did body weight (*p* < 0.001, ES = 0.96, 95% CI) and skinfold thickness (*p* < 0.001, ES = 0.97, 95% CI). The MICT group also demonstrated significant reductions in body fat (*p* < 0.001, ES = 0.76, 95% CI), body weight (*p* < 0.001, ES = 0.97, 95% CI), and skinfold thickness (*p* < 0.001, ES = 0.97, 95% CI). In contrast, the control group (CG) exhibited no statistically significant changes in any of the measured variables, with non-significant reductions observed in body fat (*p* > 0.05, ES = 0.55, 95% CI), body weight (*p* > 0.05, ES = 0.32, 95% CI), and skinfold thickness (*p* > 0.05, ES = 0.72, 95% CI). These results are visually illustrated in Figure 7, reinforcing the intervention-specific effects of structured physical training.

Figure 7 presents the CONSORT-compliant flow diagram outlining participant progression through the study. A total of 74 male adolescents were assessed for eligibility, with 14 excluded for not meeting the inclusion criteria. Sixty participants (age: 16.10 ± 0.30 years) were randomized equally into three groups: HIIT (*n* = 20), MICT (*n* = 20), and control (CG; *n* = 20). All participants received their assigned interventions, and no attrition occurred during the 8-week study. There were no losses to follow-up or discontinuations in any group. All participants were included in the final analysis, ensuring complete data retention and study integrity (54).

**Figure 8:**
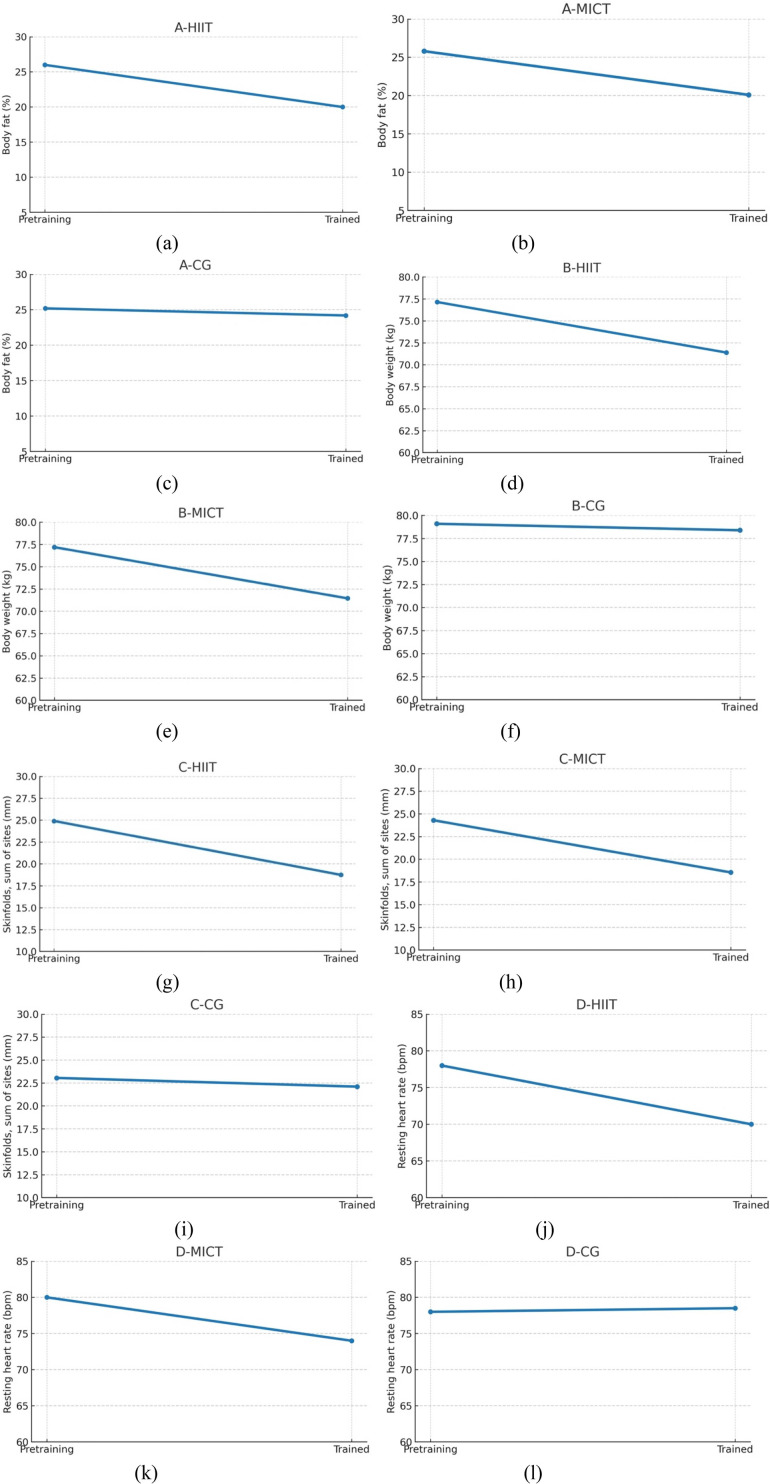
**(a–l)** post-intervention changes in body fat, body weight, skinfold thickness and resting heart rate.

Figures 8a–l present the between-group comparisons of body composition outcomes. A one-way ANOVA revealed a statistically significant difference in body fat percentage among the three groups: HIIT, MICT, and CG [F(2, 57) = 185.97, *p* < 0.001, ES = 0.86]. *post hoc* analysis indicated that all pairwise comparisons HIIT vs. MICT, HIIT vs. CG, and MICT vs. CG were significantly different, with the HIIT group showing the most pronounced reduction.

Figure 8a illustrates a clear reduction in body fat percentage among participants in the High-Intensity Interval Training (HIIT) group from the pretraining to post-training phase. Body fat decreased from approximately 26% to 20%, indicating a reduction of about 6% points over the course of the 8-week intervention. This notable decline supports the primary outcome of the study, confirming that HIIT is an effective exercise modality for reducing body fat in normal-weight sedentary male adolescents. Figure 8b illustrates the change in body fat percentage among participants in the Moderate-Intensity Continuous Training (MICT) group from the pretraining to post-training period. Body fat decreased from approximately 25.8% to 20.1%, representing a reduction of about 5.7% points over the course of the 8-week intervention. This significant decline indicates that MICT is an effective exercise strategy for reducing body fat in normal-weight sedentary male adolescents. Although the reduction was slightly less than that observed in the HIIT group, the result affirms the efficacy of MICT in improving body composition through sustained aerobic activity (55).

Figure 8c illustrates the change in body fat percentage for the Control Group (CG) from the pretraining to post-training phase. The body fat percentage decreased only slightly, from approximately 25.2% to 24.2%, reflecting a minimal reduction of about 1 percentage point over the 8-week period. This negligible change indicates that, in the absence of a structured physical activity intervention, significant improvements in body composition are unlikely. The results highlight the contrast between the intervention groups and the CG, further reinforcing the conclusion that both HIIT and MICT are effective methods for reducing body fat, while physical inactivity fails to produce similar benefits. Figure 8d illustrates the change in body weight among participants in the High-Intensity Interval Training (HIIT) group from the pretraining to post-training phase. Body weight decreased significantly from approximately 77.2 kg to 71.4 kg, reflecting a reduction of about 5.8 kg over the 8-week intervention. This substantial decline indicates that HIIT was highly effective in promoting weight loss among normal-weight sedentary male adolescents. The results support the study's sub-hypothesis that HIIT contributes meaningfully to body weight reduction and align with existing literature highlighting HIIT's capacity to induce weight loss through elevated energy expenditure, enhanced fat oxidation, and metabolic stimulation (56).

Figure 8e illustrates the change in body weight among participants in the Moderate-Intensity Continuous Training (MICT) group from the pretraining to post-training period. Body weight decreased significantly from approximately 77.2 kg to 71.5 kg, indicating a reduction of about 5.7 kg over the 8-week intervention. This substantial decline confirms that MICT is an effective strategy for promoting weight loss in normal-weight sedentary male adolescents. The extent of weight reduction is comparable to that observed in the HIIT group, suggesting that MICT can serve as a viable and sustainable method for improving body composition and managing weight when applied consistently. This finding supports the study's broader conclusion that both HIIT and MICT are effective for enhancing adolescent health outcomes.

Figure 8f shows the change in body weight among participants in the Control Group (CG) from the pretraining to post-training phase. Body weight decreased only slightly, from approximately 79.1 kg to 78.4 kg, representing a minimal reduction of about 0.7 kg over the 8-week period. This negligible change indicates that, in the absence of a structured physical activity intervention, meaningful weight loss did not occur. In contrast to the significant reductions observed in the HIIT and MICT groups, the CG results underscore the importance of consistent, planned exercise for effective weight management. This finding supports the conclusion that physical inactivity fails to produce substantial changes in body composition among sedentary adolescents.

As shown in Figure 8g, in the C-HIIT group, the summed skinfold thickness (proxy for subcutaneous adiposity) decreased from approximately 25 mm at pre-training to about 19 mm post-training, an absolute reduction of 6 mm (24%). This clear downward trend line indicates a meaningful improvement in body composition following the intervention. As shown in Figure 8h, in the C-MICT group, the summed skinfold thickness (indicator of subcutaneous adiposity) decreased from approximately 24.5 mm at pre-training to about 18.9 mm post-training, an absolute reduction of 5.6 mm (23%). This within-group decline suggests a favourable improvement in body composition following moderate-intensity continuous training. In the control group (C-CG), the summed skinfold thickness changed only marginally, from approximately 23.0 mm at pre-training to about 22.2 mm post-period—an absolute decrease of 0.9 mm (4%), as shown in Figure 8i. This small within-group shift indicates relative stability in subcutaneous adiposity over the observation interval without structured training.

Figure 8j illustrates the change in resting heart rate among participants in the High-Intensity Interval Training (HIIT) group from the pretraining to post-training period. Resting heart rate decreased notably from approximately 78 beats per minute (bpm) to 71 bpm, representing a reduction of about 7 bpm over the 8-week intervention. This significant decrease indicates improved cardiovascular efficiency and autonomic regulation, as lower resting heart rate is commonly associated with enhanced cardiovascular fitness. The result supports the study's findings that HIIT effectively promotes cardiorespiratory health in normal-weight sedentary male adolescents. It also aligns with existing research suggesting that HIIT enhances parasympathetic tone and reduces cardiovascular strain, thereby contributing to overall cardiovascular well-being.

Figure 8k illustrates the change in resting heart rate among participants in the Moderate-Intensity Continuous Training (MICT) group from the pretraining to post-training period. Resting heart rate decreased from approximately 80 beats per minute (bpm) to 74 bpm, indicating a reduction of about 6 bpm over the 8-week intervention. This meaningful decrease reflects improved cardiovascular function and autonomic regulation, as a lower resting heart rate is a well-established indicator of enhanced cardiovascular fitness. While the reduction was slightly less than that observed in the HIIT group, the result confirms that MICT is also effective in improving cardiorespiratory health in normal-weight sedentary male adolescents. These findings support MICT as a beneficial and sustainable training method for promoting heart health during adolescence.

Figure 8l illustrates the change in resting heart rate among participants in the Control Group (CG) from the pretraining to post-training period. The resting heart rate remained unchanged at approximately 78 beats per minute (bpm) throughout the 8-week duration, indicating no measurable improvement in cardiovascular function. This stability suggests that in the absence of structured physical activity, such as HIIT or MICT, no significant enhancements in resting heart rate or autonomic regulation occur. In contrast to the reductions observed in the intervention groups, this result underscores the necessity of regular exercise for promoting cardiovascular health in sedentary adolescents.

In this study, it must be stressed that baseline adiposity averaging 26% body fat appears high for adolescent males described as “normal weight.” Because BMI-for-age (kg/m^2^), height/weight z-scores, pubertal stage, or validated adiposity cut-points are not reported, the “normal-weight” classification is not verifiable and may reflect either (i) misclassification or (ii) “normal-weight obesity” (normal BMI but excess body fat). Clarifying eligibility criteria (e.g., BMI-for-age percentile range), the reference system used to judge normal weight, and the body-fat thresholds that define “excess adiposity” would resolve this concern and align the sample description with the outcomes evaluated. It was found that, the intervention effects are clear: body fat decreased from 26.0→20.0% in C-HIIT and 25.8→20.1% in C-MICT, while the control changed minimally (25.2→24.2%). Between-group ANOVA showed a large, significant effect [F(2,57) = 185.97, *p* < .001, *η*p^2^ = 0.86] with all pairwise comparisons significant and the largest reduction in HIIT. Convergent improvements were observed in surrogate adiposity measures (skinfold sums dropped by 6 mm in both training groups vs. 1 mm in controls) and body mass (4 kg decrease in HIIT/MICT vs. negligible change in controls). These findings indicate clinically meaningful reductions in adiposity with structured exercise, independent of whether participants fall into an overweight/obese category by BMI.

It was found that based on group means, the HIIT arm decreased body-fat percentage from 26.0% to 20.0% and body mass from 77.15 to 71.40 kg over 8 weeks, implying a 5.8 kg reduction in fat mass. The MICT arm declined from 25.8% to 20.1% body-fat and 77.20 to 71.45 kg, implying an 5.6 kg fat-mass reduction. Between-group differences were large [ANOVA: F(2,57) = 185.97; *η*p^2^ = 0.86], indicating substantial, time-efficient improvements in body composition achievable within a school-delivered programme. Two major findings are identified. First, body-composition estimates from BIA and anthropometry are sensitive to hydration status, device algorithms, diurnal timing, and technician variability; absolute values should be interpreted cautiously, prioritising standardised measurement conditions and change-scores over single time points. Second, the *a priori* target concerned improving body composition and cardiorespiratory fitness in sedentary adolescents. It is not indiscriminate weight loss, aligning with healthy growth and physical literacy goals. To convey clinical magnitude alongside statistical significance, responder analyses (e.g., proportions achieving ≥3 and ≥5 percentage-point reductions in body-fat) and 95% confidence intervals around mean changes (body-fat %, fat mass in kg, and body mass), together with effect sizes, should be reported.

### 3.5 Resting heart rate

The analysis of body composition outcomes before and after the training period revealed statistically significant improvements in all key metrics for the intervention groups (HIIT and MICT) compared to the control group (CG), with effect sizes across all parameters. Participants in the HIIT group experienced a significant reduction in body fat percentage from 26.00 ± 2.97% to 20.00 ± 3.21%, while those in the MICT group showed a decrease from 25.80 ± 3.03% to 20.10 ± 3.62%. In contrast, the CG exhibited only a minor reduction from 25.20 ± 3.05% to 24.20 ± 2.66%. The mean difference across groups was 4.23%, with a 95% Confidence Interval (CI) of 3.99–4.47 and a highly significant *p*-value (<0.001). The partial eta-squared (*η*p^2^) value of 0.867, affirming a strong training effect, as shown in Table 11.

**Table 11:** Parametric analysis of input variables selected for the study.

Outcomes	Group	Pretraining	Trained	*Δ* (Mean)	95% CI of *Δ*	P	(*η*p^2^)
Body Fat (%)	HIIT	26.00 ± 2.97	20.00 ± 3.21	4.23*	3.99–4.47*	0.000	0.867
	MICT	25.8 ± 3.03	20.10 ± 3.62				
	CG	25.20 ± 3.05	24.20 ± 2.66				
Body Weight (kg)	HIIT	77.15 ± 4.39	71.40 ± 4.18	4.06*	3.79–4.36*	0.000	0.846
	MICT	77.20 ± 5.01	71.45 ± 4.53				
	CG	79.10 ± 4.11	78.40 ± 3.45				
Skin caliper (mm)	HIIT	24.90 ± 2.91	18.75 ± 3.12	4.283*	4.05–4.51*	0.000	0.961
	MICT	24.3 ± 2.79	18.55 ± 3.45				
	CG	23.05 ± 3.08	22.10 ± 3.07				
Resting Heart Rate (bpm)	HIIT	78.25 ± 3.30	71.10 ± 3.07	4.283*	4.05–4.51*	0.000	0.953
	MICT	80.05 ± 3.57	74.35 ± 3.57				
	CG	78.45 ± 1.43	78.65 ± 1.98				

Values are mean (SD) unless stated. *Δ* = post−pre. MD = between-group mean difference in *Δ*. g = Hedges g (bias-corrected) with 95% CI**.** partial ηp^2^ with 95% CI computed from the non-central F distribution. Exact *p*-values shown; qualitative effect labels are not used.

*Significant values.

The HIIT group reduced their body weight from 77.15 ± 4.39 kg to 71.40 ± 4.18 kg, while the MICT group showed a similar decline from 77.20 ± 5.01 kg to 71.45 ± 4.53 kg. The control group demonstrated only a negligible change, decreasing from 79.10 ± 4.11 kg to 78.40 ± 3.45 kg. The overall mean reduction was 4.06 kg, with a 95% CI of 3.79–4.36 (*p* < 0.001), and an *η*p^2^ value of 0.846.

Skinfold calliper values decreased notably in both intervention groups: from 24.90 ± 2.91% to 18.75 ± 3.12% in the HIIT group, and from 24.30 ± 2.79% to 18.55 ± 3.45% in the MICT group. The CG exhibited a marginal decrease from 23.05 ± 3.08% to 22.10 ± 3.07%. The mean difference was 4.283%, with a 95% CI of 4.05–4.51, a *p*-value < 0.001, and an effect size of *η*p^2^ = 0.961, which is classified as very large, confirming substantial differences attributable to the intervention. Resting heart rate significantly improved in the HIIT group (from 78.25 ± 3.30 bpm to 71.10 ± 3.07 bpm) and the MICT group (from 80.05 ± 3.57 bpm to 74.35 ± 3.57 bpm). The CG saw a negligible increase from 78.45 ± 1.43 bpm to 78.65 ± 1.98 bpm. The average difference across groups was 4.283 bpm (95% CI: 4.05–4.51), with *p* < 0.001 and an effect size of *η*p^2^ = 0.953.

In summary, body fat percentage decreased substantially in both intervention groups relative to control. HIIT fell from 26.00% to 20.00% and MICT from 25.80% to 20.10% (−5.70 percentage points), whereas the control group declined modestly from 25.20% to 24.20%. Between-group differences were significant with ANOVA: F(2,57) = 185.97, *p* < .001, *η*p^2^ = 0.86), and all pairwise comparisons were significant. The overall pooled mean reduction across groups was approximately 4.23% (95% CI 3.99–4.47), with HIIT yielding the largest decrease despite shorter session durations.

Secondary outcomes showed consistent, clinically meaningful improvements with training. Body weight declined by 5.75 kg in both HIIT (77.15→71.40 kg) and MICT (77.20→71.45 kg), compared with a smaller change in controls (79.10→78.40 kg); the between-group effect was large [F(2,57) = 157.06, *p* < 0.001, *η*p^2^ = 0.84], and the mean reduction across groups was 4.06 kg (95% CI 3.79–4.36). Skinfold-derived adiposity decreased markedly in the training arms (HIIT 24.90→18.75%; MICT 24.30→18.55%) vs. a minor reduction in controls (23.05→22.10%), with a very large group effect [F(2,57) = 210.95, *p* < 0.001, *η*p^2^ = 0.96]. Resting heart rate also improved, falling by 7.15 bpm with HIIT (78.25→71.10 bpm) and 5.70 bpm with MICT (80.05→74.35 bpm), while remaining essentially unchanged in controls (78.45→78.65 bpm); the effect size was again very large (*η*p^2^= 0.95; *p* < 0.001).

It must be stressed that both HIIT and MICT produced large and clinically meaningful benefits relative to no structured exercise, spanning reductions in body fat, body weight, and skinfold percentage, alongside improvements in resting heart rate. Notably, HIIT achieved comparable and in some outcomes slightly greater improvements than MICT with approximately one-third shorter sessions, underscoring its suitability as a time-efficient modality for integration into school physical education. Furthermore, no adverse events were observed or reported during training or testing across the 8-week intervention. In this study, it was found that both HIIT and MICT produced substantial improvements in adiposity and cardiovascular fitness relative to control. There were no injuries requiring first aid or medical evaluation, no episodes of syncope, chest pain, respiratory distress, or heat illness, and no session terminations or withdrawals attributable to harms; retention remained 100% in all groups.

## 4 Discussion

### 4.1 Interpretations of outcomes

This randomized, school-based trial in sedentary, normal-weight adolescent males found modest improvements in adiposity and resting heart rate across the exercise arms, but the planned manipulation check revealed little physiological separation between the prescribed 65% and 85% sessions, indicating fidelity issues in achieving target intensity. In the broader adolescent literature, an 8-week head-to-head trial similarly reported that HIIT and MICT both reduced body fat mass, body-fat percentage, and visceral fat area, with within-group reductions in systolic/diastolic blood pressure and triglycerides observed only in HIIT suggesting that, when delivered as intended, both modalities can move body composition favourably, and HIIT may confer additional cardiometabolic advantages (3). It was found that higher-intensity intervals can augment post-exercise lipid metabolism. In an isocaloric crossover, HIIT produced greater excess post-exercise oxygen consumption and higher post-exercise lipid oxidation than continuous running, despite matched exercise energy expenditure—an effect most pronounced early in recovery (57). These findings support the plausibility that, once intensity is achieved, HIIT can amplify fat-loss signals beyond MICT. Time-efficiency central to school implementation is also supported by adult data: a 6-week RCT showed comparable overall training effects across HIIT and MICT, yet the percentage change favoured HIIT for VO₂ max and trended toward greater fat-mass reduction, despite shorter sessions, aligning with the rationale for higher-intensity, lower-time prescriptions (21). In this study, the measurement battery did not include maximal aerobic capacity or clinical cardiometabolic markers; therefore, causal statements and claims about long-term or clinical risk modification cannot be drawn. To strengthen future inference, studies should incorporate criterion or higher-fidelity endpoints such as directly measured VO₂max or 20-m shuttle-derived ^C^V̇O_2_ peak, blood pressure, and additional cardiometabolic markers alongside the present anthropometric indices, thereby enabling conclusions that extend beyond fitness surrogates to cardiovascular function and health. In adolescents, both HIIT and MICT are associated with improvements in adiposity and cardiovascular fitness, with HIIT frequently yielding greater increases in aerobic capacity and selected cardiometabolic markers (3, 17, 58).

The present manipulation-check shortfall highlights the importance of objective dosing and progressive overload. Prescribing and re-programming workloads from functional anchors elicit clinically meaningful responses; intensities below this threshold typically do not (59). Operationally, progressive re-adjustment of workloads across weeks is “fundamental” to generate improvement guidance that maps directly onto school-based scheduling and supervision needs (59). In this study, feasibility and safety for supervised high-intensity work are well-documented: in a multicentre RCT, treadmill HIIT improved 6-min walk distance, balance, and executive function after the intervention, with some cognitive benefits persisting at 12 months (6). Although clinical and adult, these data reinforce that high-intensity, heart-rate-anchored protocols can be implemented effectively when supervision and monitoring are robust precisely the conditions that should be emphasized in school settings. Align the program with verified intensity attainment to realize the theoretical advantages seen in controlled trials; when fidelity is ensured, both HIIT and MICT improve adolescent body composition, with HIIT offering potential added cardiometabolic and time-efficiency benefits (3, 21, 57).

The superiority of HIIT over MICT can be partly explained by acute potentiation phenomena that bias recruitment toward type II fibers and transiently elevate contractile performance after vigorous bouts. Recent syntheses differentiate post-activation potentiation (PAP) from post-activation performance enhancement (PAPE): both enhance force output with a short delay and show larger effects in fast-twitch–dominant musculature, but PAPE is more strongly influenced by increased muscle temperature and intramuscular fluid shifts, whereas PAP is classically linked to myosin regulatory light-chain (RLC) phosphorylation and heightened Ca^2+^ sensitivity at the cross-bridge (60). These PAP/PAPE responses most pronounced in type II fibers are precisely the fibers preferentially engaged by HIIT's intense work intervals, thereby offering a physiologically coherent pathway by which HIIT can acutely raise rate-of-force development and economy in subsequent efforts within a session (60). While neural contributions are less consistent, the literature notes possible but not uniformly demonstrable neurogenic influences on PAPE, again consistent with HIIT's repeated high-intensity contractions (60).

In this study, these mechanisms provide a plausible explanation for the slightly larger cardiovagal adaptation observed with HIIT (resting heart rate −7.15 bpm) compared with MICT (−5.70 bpm), alongside comparable or marginally greater reductions in adiposity achieved in one-third less time per session. Consistent with these patterns, our pre- to post-data show body-fat percentage decreasing from 26.0→20.0% with HIIT and 25.8→20.1% with MICT, with very large between-group effects, despite equal total weekly frequency (4×/week). Although classic PAP/PAPE are acute, repeated exposure to the underlying triggers of fast-fiber recruitment and elevated Ca^2+^/metabolic signalling during HIIT plausibly scales into chronic adaptations, such as improved autonomic balance, higher glycolytic/oxidative enzyme activity, and better movement economy thereby helping HIIT match or exceed MICT's outcomes with less time burden.

The broader student health context reinforces the value of high-intensity work. In university students, higher physical activity particularly at greater intensities shows positive associations with academic performance, with cognition mediating part of this relationship (61). While our study focused on physiological endpoints, these findings suggest that HIIT's briefer, potent stimuli may confer additional neurocognitive advantages relevant to school settings, complementing its efficiency and cardiometabolic benefits.

Notably, youth-specific detraining evidence highlights why targeting fast-twitch function and rapid force expression properties potentiated within HIIT matters. After pandemic-related activity restriction, young adults exhibited higher fat percentages and sex-specific decrements in isometric strength, highlighting the vulnerability of neuromuscular capacity to reduced activity and the potential need for intensity-rich programming to restore function (62). In practice, structuring school-based HIIT to respect PAPE timing (minutes-scale recovery between intense sets) while ensuring sufficient warm-up to leverage temperature- and fluid-mediated enhancements can help translate these molecular and physiological mechanisms into reliable improvements across a term (60).

In this randomised, school-based trial in normal-weight adolescent males, both HIIT and MICT substantially improved body composition and resting heart rate relative to control. Notably, HIIT achieved comparable or significantly greater benefits in less time, supporting its time-efficiency for PE timetables and addressing a key translational barrier in adolescent exercise programming.

It was found that across 8 weeks, HIIT and MICT produced 5–6 percentage-point reductions in whole-body fat, corresponding to an estimated 5.6–5.8 kg decrease in fat mass based on group means. In sedentary, otherwise normal-weight adolescents, such shifts indicate meaningful improvements in body composition achieved within school-compatible schedules. While bioelectrical impedance and skinfolds have known error bounds, convergent reductions across %BF, skinfolds, and body mass and the large between-group effects support the practical significance of the intervention. In school settings, HIIT may be a potentially time-efficient alternative when comparable adaptations are achieved within shorter planned sessions, as observed in adolescent interventions (3, 58)

The comparative quantitative profile indicates that the present school-based RCT in normal-weight adolescent males achieved large, convergent improvements in adiposity and cardiovascular fitness over 8 weeks body-fat percentage decreased by 6.0 (HIIT) and 5.7 percentage points (MICT), body mass fell by 5.8 kg in each training arm, summed skinfolds declined by 23%–25%, and resting heart rate dropped by 7 bpm (HIIT) and 6 bpm (MICT), each with very large between-group effects vs. control. These magnitudes substantially exceed the body-composition contrasts typically observed between training modalities in meta-analytic youth syntheses, which report little or no superiority of HIIT over alternative exercise for fatness indices but clear advantages for aerobic capacity and systolic blood pressure. The García-Hermoso et al. (33) meta-analysis in overweight/obese youth found HIIT produced greater increases in VO₂max (SMD = 0.59; + 1.9 ml kg^−1^·min^−1^) and larger reductions in systolic blood pressure (WMD = −3.6 mmHg) than comparison exercise, while differences in fat mass, BMI and waist circumference were null. The divergence suggests that, although interval formats are consistently advantageous for cardiorespiratory adaptation and blood pressure, body-composition change may depend more on total loading, energy balance, and population characteristics than on intensity pattern *per se*; the present trial's sizeable adiposity shifts therefore likely reflect high total stimulus and tight delivery within a supervised school context rather than a modality effect alone.

Methodological contrasts also qualify interpretation. The meta-analysis prioritised VO₂max and clinical markers (blood pressure, lipids, insulin indices) and demonstrated that HIIT's superiority strengthens in studies ≥12 weeks, whereas the present 8-week trial indexed cardiovascular fitness primarily via resting heart rate and did not collect direct clinical risk markers, limiting claims to fitness rather than health. Moreover, the trial's fidelity checks revealed minimal physiological separation between prescribed intensities across sessions, indicating a failed manipulation check that complicates attribution of effects to “high-” vs. “moderate-” intensity dosing an issue not evident in the aggregated trials where VO₂max gains clearly favoured HIIT over MICT comparators. Finally, body composition was assessed with BIA and skinfolds in the trial, methods the meta-analysis explicitly cautions can vary in accuracy relative to criterion techniques in paediatric samples; this underscores the importance of emphasising change scores under standardised conditions while avoiding over-interpretation of absolute values. To sum up, the evidence base supports the manuscript's central translational claim school-embedded HIIT can match MICT with less time yet indicates that uniqueness lies in the normal-weight, male, school setting and the magnitude of adiposity change rather than in demonstrating HIIT's categorical superiority on clinical cardiometabolic outcomes. Furthermore, the observed pre- to post-intervention differences support an association between structured exercise and improvements in adiposity and cardiovascular fitness (indexed only by resting heart rate), but they do not establish durable or clinical effects in the absence of follow-up or direct health markers (e.g., blood pressure, lipid profile). Fidelity analyses indicate limited physiological separation between the prescribed intensity conditions across sessions, which complicates attribution of effects to “high-” vs. “moderate-” intensity *per se*. Additionally, potential confounding from unquantified dietary intake and reliance on BIA/skin-fold methods such sensitive to hydration and technician variability. The study further argues for cautious interpretation focused on standardized change scores rather than strong causal or clinical claims.

A comparative study highlights that both trials report favourable changes in adiposity, yet their quantitative profiles and populations differ in ways that temper cross-study inference. In normal-weight adolescents over 8 weeks, Sun et al. (3) observed similar within-group reductions in body fat mass, body-fat percentage, and visceral fat area in HIIT and MICT, with no between-group differences on clinical biomarkers; notable within-group declines emerged for systolic/diastolic blood pressure and triglycerides only in the HIIT arm (e.g., SBP *p* = 0.018, ES = 0.84; DBP *p* = 0.008, ES = 1.76; TG *p* = 0.004, ES = 1.33) despite the small sample (*n* = 18, 3 days·week^−1^) and short duration. These findings suggest comparable body-composition responsiveness to both modalities under school-compatible dosing, with possible added cardiometabolic reactivity to higher intensities, but the absence of VO₂-based fitness outcomes and limited power constrain interpretation of modality superiority. Furthermore, adherence was reported as 100% in both intervention arms, reducing concerns that differential compliance inflated effects, yet external validity remains bounded by the single-site, short-term design.

By contrast, Meng et al. (58) extended training to 12 weeks in obese boys and quantified aerobic capacity, showing significant BMI and fat-mass reductions in both groups, a larger VO₂peak gain with HIIT than MICT (+6.1 vs. + 3.8 ml kg^−1^ min^−1^), and selective lipid and insulin-resistance improvements (LDL decreased only after HIIT; HOMA-IR improved in both). The protocol also demonstrated pragmatic efficiency 11-min HIIT sessions achieved composition and cardiovascular fitness changes comparable to 30-min MICT though differences in age, adiposity, and program length limit direct comparison to the normal-weight cohort. To sum up, the obese-youth trial supports a quantitative advantage of HIIT for aerobic capacity and LDL alongside broad adiposity benefits in both arms, whereas the normal-weight trial indicates parity in adiposity outcomes with hints of greater blood-pressure and triglyceride responsiveness to HIIT. The convergent message is that, across distinct populations, HIIT and MICT both yield meaningful improvements, with HIIT showing potential advantages in cardiovascular fitness and selected cardiometabolic markers under longer dosing or in higher-risk cohorts an interpretation that highlights the need for adequately powered, VO₂-anchored, and longer-term school-based comparisons in normal-weight adolescents.

### 4.2 Limitations of the study

The sample comprised only male adolescents, limiting generalizability to females. During adolescence, rapid endocrine remodelling by rising testosterone and growth hormone in boys and cyclic fluctuations of estrogen and progesterone in girls can differentially shape training responses. In males, pubertal increases in anabolic hormones support gains in fat-free mass, erythropoiesis/haemoglobin mass, and aerobic capacity potential, which may amplify adaptations to high-intensity stimuli relative to age-matched females. Maturation status also modulates exercise endocrine responses (e.g., larger growth-hormone responses in pubertal vs. pre-pubertal cohorts), implying that HIIT's high metabolic stress could preferentially enhance adaptations in mid- to late-pubertal boys. In this study, the trial's duration (8 weeks), single-sex cohort, single setting, lack of clinical cardiometabolic endpoints, and absence of post-intervention follow-up constrain causal and external validity. Accordingly, statements implying sustained or clinical benefits should be reframed to emphasize improved cardiovascular fitness and adiposity over the study window, acknowledge the failed intensity manipulation check, and indicate that confirmation of durability, dose–response, and clinical translation requires longer trials with verified intensity attainment and direct health markers.

By contrast, females often exhibit greater reliance on lipid oxidation at a given relative intensity and experience menstrual-cycle–related shifts in substrate use, thermoregulation, and perceived exertion factors that may alter both acute responses and training adaptations to HIIT vs. MICT across the cycle and increase within-group variability. Cycle-phase variation in wellness and injury risk among adolescent female athletes may further interact with training dose and recovery. Evidence from youth cohorts also indicates sex-specific patterns in body-composition and strength trajectories, highlighting the need for sex-inclusive designs (62). In this study, because only resting heart rate was collected as a cardiovascular outcome, references to cardiovascular health were revised to cardiovascular fitness to avoid overstating clinical implications. Although random assignment and allocation concealment were implemented, the absence of objective monitoring in the control arm and the lack of quantified dietary intake introduce potential residual confounding. Incomplete fidelity capture in MICT further limits precision in between-arm contrasts. Accordingly, findings are best framed as short-term associations within a school-based randomized design; future trials should incorporate device-based activity tracking for all arms, quantified dietary assessment, and complete session adherence/fidelity records to enhance internal validity.

Notably, these hormonal and maturational differences suggest that the magnitude and pattern of changes in adiposity, resting heart rate, and cardiorespiratory fitness observed in the present study may not directly extrapolate to mixed-sex cohorts and could differ among adolescent girls. Future investigations should include female participants; stratify or adjust for pubertal stage; in female arms, standardize testing and key HIIT sessions by menstrual-cycle phase and contraceptive use; and consider measuring haemoglobin mass/iron status and relevant hormones to strengthen mechanistic inference (63). Furthermore, linking HIIT session design to potentiation mechanisms—e.g., timing rest intervals to leverage post-activation performance enhancement may help explain and compare sex-specific responses in adolescent samples (60–62).

Body composition outcomes were indexed by body mass and summed skinfolds, and cardiovascular fitness by resting heart rate. These indices are useful but indirect surrogates: body mass conflates fat and fat-free compartments; skinfolds estimate subcutaneous rather than total or visceral adiposity; and resting heart rate reflects autonomic balance and training status rather than maximal aerobic capacity or vascular function. For future trials aimed at clinical or mechanistic claims, add criterion or higher-fidelity measures (e.g., VO₂max or 20-m shuttle-derived peak, blood pressure, fasting lipids/glucose, heart-rate variability, or echocardiographic/flow-mediated dilation where feasible) and a multi-compartment body-composition method (DXA or BIA with standardized hydration checks) to triangulate adiposity change.

### 4.3 Practical implications and educational guidelines

The study findings highlight that HIIT is well suited to school timetables because it yields comparable or greater benefits than MICT in less time and requires minimal equipment. Within a 35–40-min lesson, a compact 20-min block works effectively: 5–6 min of warm-up (light jog, dynamic mobility), a 12–13-min main set of six 30-s efforts at 80%–90% HRmax interleaved with 90-second easy walking (50% HRmax), and a 3–4-minute cool-down. For 45–50-min lessons, a mixed-fitness option can extend the main set to 16–18 min using 8–10 cycles of 30 s hard with 60–90 s easy. Space constraints can be managed by marking lanes or shuttle distances with cones and staggering pods by 30–60 s.

For weekly scheduling, aim for two HIIT lessons during PE, with more than 24 h between sessions; an optional extracurricular HIIT block can be added when feasible. Intensity can be prescribed with heart-rate monitors (80%–90% HRmax work, 50%–60% recovery) or, where unavailable, with RPE (8–9/10 hard; 3–4/10 easy) and the talk test. As a fidelity cue, target more than 80% of each work interval within the intended zone.

Progress over 8 weeks with small, manageable changes: Weeks 1–2, 6 × 30 s/90 s; Weeks 3–4, 8 × 30 s/75 s; Weeks 5–6, 8–10 × 30 s/60 s; Weeks 7–8, 10 × 30 s/60 s or maintain eight rounds while increasing distance per 30-second effort. Maximize time-on-task with rapid set-up, staggered starts, and a simple whistle cadence. Use inclusive A/B/C distances so all students reach target intensity; apply routine warm-up and cool-down and follow school health clearance procedures. To track impact with teacher-friendly measures, record resting heart rate, body mass, and a simple fitness marker before and after a 6–8-week unit.

Monitoring training load and recovery is essential during adolescence, when growth and maturation can alter readiness and tolerance to exercise from week to week. In this study, external load and intensity were anchored to heart-rate reserve with continuous HR capture at 5-s epochs, compliance was defined as more than 80% time-in-zone, sessions were scheduled four times weekly with more than 24 h between sessions, and delivery was supervised providing a strong baseline for load governance. The manipulation check indicated poor physiological separation between the prescribed intensities, suggesting that additional internal-load and recovery indices would improve fidelity and participant safety (e.g., pairing HR/time-in-zone with session-RPE, and using brief wellness screens for sleep, soreness, and stress). For school implementation, PE staff can individualize daily targets by combining (i) objective metrics %HRR time-in-zone and completion of planned reps with (ii) simple subjective markers session-RPE (0–10), morning fatigue, and muscle soreness to trigger modifications (longer recoveries, reduced repetitions, or a light day) when recovery is compromised. A weekly dashboard (total sessions, time-in-zone, mean session-RPE, and training monotony/strain) plus preplanned reload weeks can mitigate cumulative fatigue while preserving the demonstrated benefits of HIIT/MICT in this population. These procedures are consistent with the supervised, HR-guided delivery used here and are feasible with minimal equipment in school settings. In this study, findings extend meta-analytic conclusions by demonstrating that, in a normal-weight male school cohort, both HIIT and MICT elicit large, parallel improvements in adiposity and cardiovascular fitness, with HIIT achieving similar benefits in one-third less session time. This school-embedded, teacher-supervised model directly addresses implementation barriers highlighted by prior reviews and offers an operational template session length, frequency, and monitoring that can be adopted within typical PE timetables.

## 5 Conclusions

Across eight weeks, both HIIT and MICT produced large, clinically meaningful improvements in adiposity and cardiorespiratory fitness relative to control, as evidenced by significant reductions in resting heart rate. Body-fat percentage fell from 26.00% to 20.00% with HIIT (−6.00 percentage points) and from 25.80% to 20.10% with MICT (−5.70 points); between-group differences were large [ANOVA: F(2,57) = 185.97, *p* < .001, *η*p^2^ = 0.86]. Body mass decreased by ∼5.75 kg in both HIIT (77.15→71.40 kg) and MICT (77.20→71.45 kg), again with a large between-group effect [F(2,57) = 157.06, *p* < .001, *η*p^2^ = 0.84]. Skinfold sums dropped from 24.90 to 18.75 mm (HIIT) and 24.30 to 18.55 mm (MICT), accompanied by (*η*p^2^ = 0.961). Resting heart rate improved from 78.25 to 71.10 bpm (HIIT) and 80.05 to 74.35 bpm (MICT). Notably, HIIT delivered benefits comparable to MICT despite shorter sessions (20 vs. 30 min), supporting its time-efficiency for school settings.

This study provides evidence that a brief, HRR-prescribed, teacher-deliverable HIIT protocol (six × 30-s efforts with 90-s walking; 20 min total) matches a longer continuous MICT session (30 min) for reducing adiposity and resting heart rate in adolescent males. By quantifying changes in body fat, body mass, skinfolds, and resting heart rate alongside effect sizes and confidence intervals, the study strengthens the case for integrating short-duration HIIT blocks into PE timetables without sacrificing efficacy.

## Data Availability

The raw data supporting the conclusions of this article will be made available by the authors, without undue reservation.

## Appendix A – Dietary Plan

**Table:**

Meal	Time	Foods & portions
Breakfast	07:00–08:00	• 1 boiled egg or omelette/scrambled eggs made from 1 egg (in 1 tsp olive oil) • 2 slices light white cheese or 2 slices low-salt halloumi • Tomatoes, cucumbers, greens, lettuce, etc. (unlimited) • 5–6 low-salt olives or 2 walnuts • 2 slices whole-grain bread or 4 plain rusks
Snack 1	10:00–10:30	• 1 fresh fruit (e.g., 1 medium pear or peach) **+** about 1 small coffee cup of unsalted roasted chickpeas
Lunch	12:00–13:00	• 100 g meat: grilled/boiled chicken or grilled meat/chicken meatballs (3 meatball-size pieces) • 4 Tbsp vegetable dish (e.g., green beans, zucchini, broccoli) • Large green salad (unlimited) with 1 tsp olive oil + apple vinegar • 2 Tbsp light yogurt • Choose one: 2 slices whole-grain bread or 6 Tbsp bulgur/rice pilaf or 3 ladles whole-grain pasta (or 1 slice whole-grain bread **+** 4 Tbsp cooked legumes)
Snack 2	15:00–16:00	• 1 fresh fruit (e.g., 1 medium pear or peach) **+** 7–8 raw almonds or 2 whole walnuts
Dinner	18:00–19:00	• 100 g meat: grilled/boiled chicken or grilled meat/chicken meatballs (3 meatball-size pieces) • 4 Tbsp vegetable dish (e.g., green beans, broccoli) • Large green salad (unlimited) with 1 tsp olive oil + apple vinegar • 2 Tbsp light yogurt • 2 slices whole-grain bread
Evening snack	21:00–22:00	• 1 fresh fruit (e.g., 1 kiwi or 1 medium apple/pear) **+** 2 grissini (breadsticks)
